# Biocompatibility of 3D-printed vs. thermoformed and heat-cured intraoral appliances

**DOI:** 10.3389/fbioe.2024.1453888

**Published:** 2024-10-29

**Authors:** Joanna Weżgowiec, Andrzej Małysa, Wojciech Szlasa, Julita Kulbacka, Agnieszka Chwiłkowska, Marek Ziętek, Mieszko Więckiewicz

**Affiliations:** ^1^ Department of Experimental Dentistry, Faculty of Dentistry, Wroclaw Medical University, Wroclaw, Poland; ^2^ Department of Molecular and Cellular Biology, Faculty of Pharmacy, Wroclaw Medical University, Wroclaw, Poland; ^3^ Department of Immunology and Bioelectrochemistry, State Research Institute Centre for Innovative Medicine, Vilnius, Lithuania

**Keywords:** additive manufacturing, biomedical and dental materials, oral device, occlusal splint, dental resins, polymer, fibroblasts, cytotoxicity

## Abstract

**Objectives:**

The development of additive manufacturing has the potential to revolutionize the fabrication of medical devices. This technology, also known as 3D printing, offers precise, cost-effective, and personalized approaches, which could be particularly beneficial in the production of intraoral appliances. Despite its promise, research on the biocompatibility of 3D-printed intraoral devices is still limited. Our study aims to address this gap.

**Methods:**

We examined the cytotoxicity of materials processed via three techniques commonly used for the fabrication of different intraoral appliances: 3D printing (Dental LT Clear), thermoforming (Duran adjusted with Durasplint LC), and conventional heat-curing (Villacryl H Plus). We also investigated the impact of chemical or UVC disinfection on the biocompatibility of these materials. We assessed the biological effects induced in human gingival fibroblasts (HGFs) through both direct contact tests (MTT and LDH assays) and extract tests (PrestoBlue, DCF, and cell death type assays). Additionally, we observed changes in cellular morphology and migration rate under an inverted light microscope. The surface roughness of materials was evaluated using contact profilometry. Statistical analysis was conducted using two-way analysis of variance.

**Results:**

Our findings suggest that all three fabrication techniques induced a slight cytotoxic effect in HGFs, as evidenced by both direct contact and extract tests. However, these materials could be considered nontoxic according to the ISO 10993-5:2009 norm, as the decrease in metabolic activity observed was always less than 30% compared to the untreated control.

**Conclusion:**

This novel study confirms that 3D printing may be a safe alternative to conventional methods for fabricating intraoral appliances. However, further tests assessing the long-term intraoral usage are still needed.

## Graphical Abstract

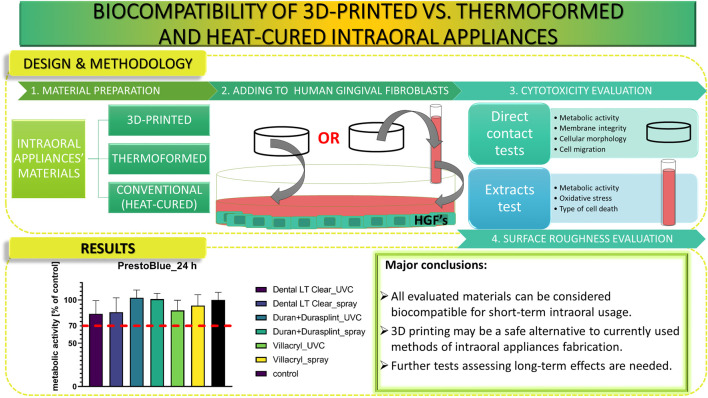

## 1 Introduction

Modern dentistry has embraced 3D printing for various applications, including the fabrication of dental casts, individual surgical guides, custom impression trays, orthodontic appliances, implants, temporary crowns and bridges, denture bases and teeth, and occlusal splints. The huge potential of this technology could be particularly useful for the rapid manufacturing of various kinds of personalized intraoral appliances since their popularity is still growing due to increasing life expectancy and aesthetic requirements (Ahmad et al., 2022; Paradowska-Stolarz et al., 2023; Chang et al., 2021; Sun et al., 2024; Zhao et al., 2024; Chen et al., 2023).

Currently, traditional methods of intraoral appliance manufacturing, which rely on hand-processed acrylic resins (heat-cured or self-cured) or thermoformed materials, are still widely used despite being time-consuming (Alajbeg et al., 2020). Its widespread use may result from the long tradition and the concerns related to the application of newer materials, which were not tested thoroughly enough, thus the possible biological risks associated with them remain unknown. However, the adoption of digital workflows, including both subtractive and additive approaches, has the potential to streamline the process of the production of intraoral appliances, enhancing efficiency and precision while reducing the time required for fabrication. Additive manufacturing also presents the advantage of reducing materials waste (Benli et al., 2023).

Undoubtedly, practical aspects of convenience should not be prioritized over other important features that guarantee proper functioning, especially for devices intended for long-term direct contact with oral tissues. Therefore, attention must be paid to the biological risks associated with intraoral appliances (Qin et al., 2024). The biocompatibility of novel materials used in additive manufacturing for these appliances is currently undergoing extensive investigation, but further research is still needed for a complete understanding (Da Silva et al., 2023; Goracci et al., 2023; Bieger et al., 2023; Schubert et al., 2021; Raszewski et al., 2022). In particular, the number of studies using a standardized approach to compare the cytotoxicity of various materials in an easily reproducible way is still limited. Moreover, most of the studies are concerned with *in vitro* research, while clinical investigations are rare. Despite the great potential of 3D printing technology, increasing its clinical applicability is still challenging due to some problems, such as material cost, time-consuming post-processing, and the lack of well-trained personnel. It could be expected that a detailed evaluation of the properties of new materials intended for this technology would also improve its applicability (Tian et al., 2021; Balhaddad et al., 2023).

The aim of this *in vitro* study was to compare the biocompatibility of materials processed using three different techniques for the fabrication of intraoral appliances: 3D printing, thermoforming, and conventional heat-curing. This study builds upon our previous research, which focused on comparing the mechanical properties of these materials (Weżgowiec et al., 2024). Our current study provides valuable insight into the specific cellular effects of the studied materials, apart from a typical evaluation of basic changes of viability. Moreover, detailed biological effects were assessed via both direct contact and extract tests, enabling comprehensive analysis. The current investigation aimed to test the hypothesis that all studied materials processed via these three different technologies and subjected to chemical or Ultraviolet-C (UVC) disinfection would not induce a significant cytotoxic effect in human gingival fibroblasts.

## 2 Materials and methods

### 2.1 Materials, study design, and specimen preparation

**FIGURE 1:**
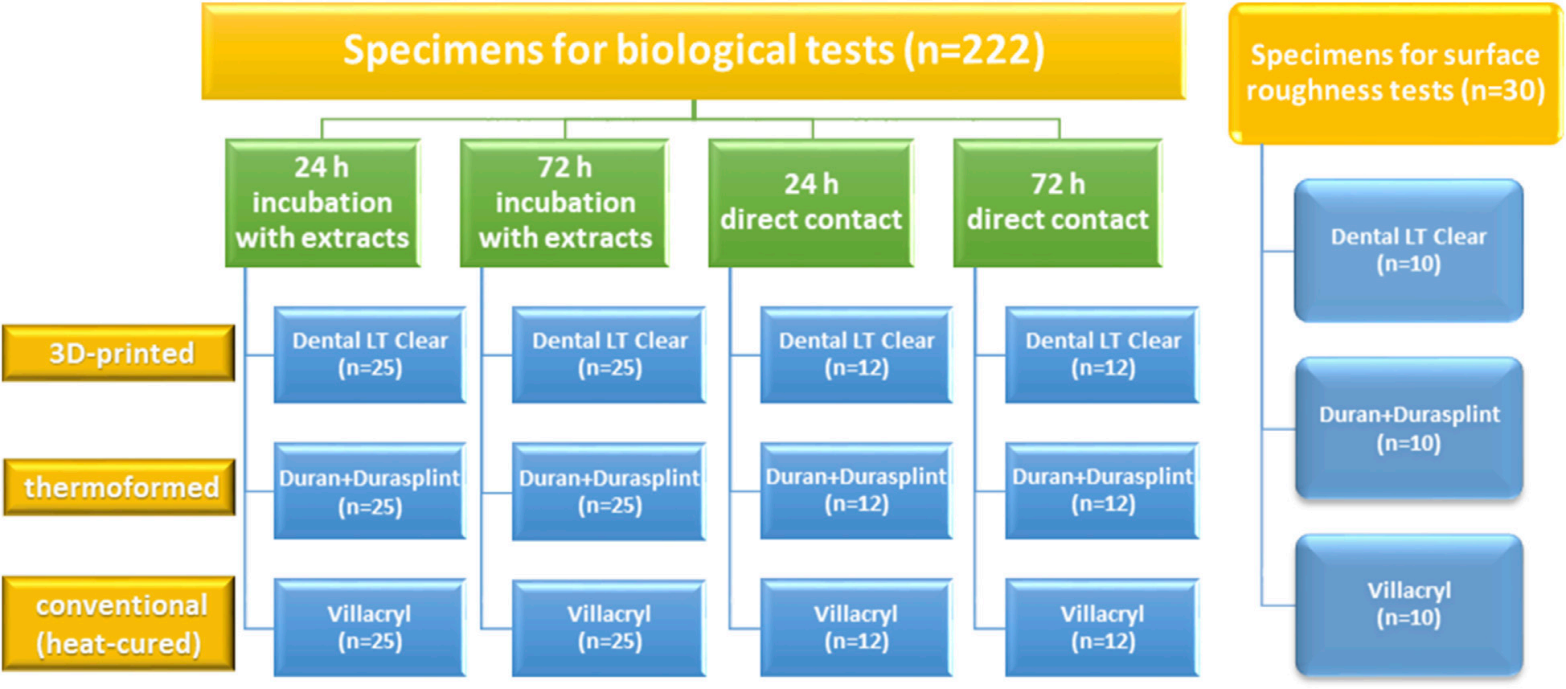
Design of the study illustrates the study groups included.

The study design is illustrated in Figure 1. The materials evaluated include (1) a 3D-printable photopolymer resin (Dental LT Clear, Vertex Dental, Soesterberg, Netherlands), (2), a thermoformable Polyethylenterephthalat-Glycol Copolyester (PET-G) foil (Duran, SCHEU-DENTAL GmbH, Iserlohn, German) with a built-up made of a light-curing mixture of acrylic resins (Durasplint LC, SCHEU-DENTAL GmbH), and (3) a conventional hand-processed heat-curing acrylic resin (Villacryl H Plus 0, Everall7, Warsaw, Poland).

**FIGURE 2:**
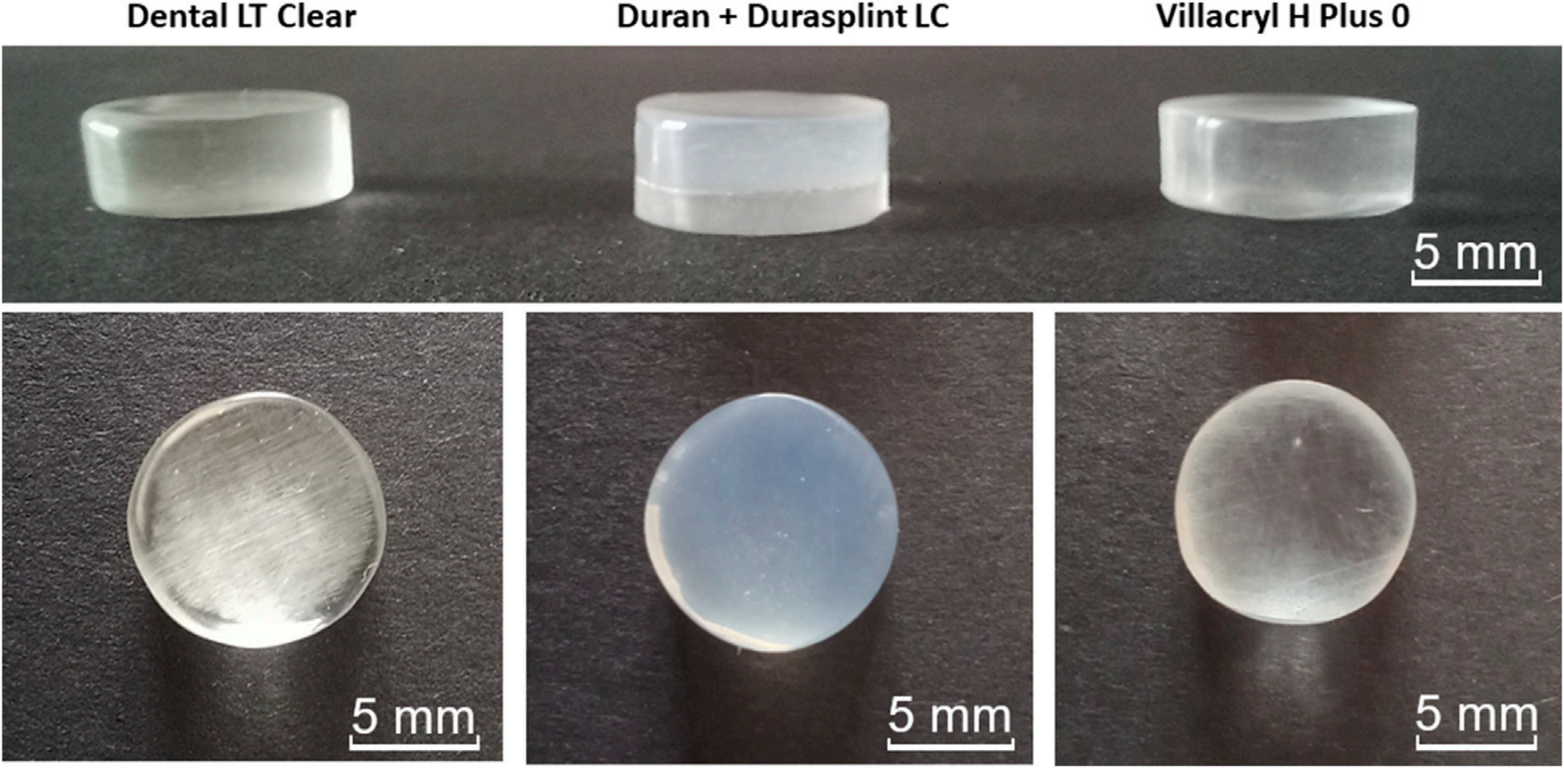
Disc-shaped specimens were manufactured via 3D printing (Dental LT Clear), thermoforming (Duran + Durasplint LC), and heat-curing (Villacryl H Plus 0).

Disc-shaped specimens with a diameter of 10 mm and a height of 4 mm were prepared for each material following the manufacturer’s recommendations, as depicted in Figure 2.

#### 2.1.1 3D printing

The specimens made of Dental LT Clear resin were designed using Meshmixer ver. 3.5.474 (Autodesk, San Francisco, CA, United States). Subsequently, PreForm software ver. 3.28.1 (Formlabs Inc., Somerville, MA, United States) was used to add supports and set printing parameters. The specimens were printed using a Form 2 printer (Formlabs) at a resolution of 100 μm, with layers oriented at 90° to the building platform. After printing, the specimens were washed 2 min × 10 min in 99% isopropanol (PPH STANLAB, Lublin, Poland) and air-dried at room temperature (RT) for 30 min. They were then postcured in a Form Cure (Formlabs) at 80°C for 20 min. Finally, the supports were removed, and the specimens were finished as described below.

#### 2.1.2 Thermoforming

The specimens made of 1.5 mm Duran were thermoformed using the pressure molding unit Ministar S (SCHEU-DENTAL GmbH). The base was covered with a thin layer of LC-Primer (SCHEU-DENTAL GmbH) and polymerized for 5 min in an LC-6 Light Oven (SCHEU-DENTAL GmbH) adapted with a 2.5 mm layer of Durasplint LC. Finally, the specimens were cured for 2 min × 10 min using the LC-6 Light Oven and were finished as described below.

#### 2.1.3 Conventional heat curing

The specimens made of Villacryl H Plus 0 were manually processed by mixing 120 g of powder with 50 g of liquid. After 25 min, when the material reached the dough-like stage, it was placed into a gypsum mold made of GC Fujirock EP (GC Europe, Leuven, Belgium) in a polymerizing flask. The material was then pressed for 15 min under 8.6 bar using a P-400 hydraulic press (Sirio Dental, Meldola FC, Italy). Finally, it was polymerized in a polymerization unit (IS-P1; InterSonic, Olsztyn, Poland) under short-term conditions, including 30 min in water heated from 60°C to 100°C, followed by 30 min at 100°C, and then 30 min at RT. Finally, the specimens were finished as described below.

#### 2.1.4 Finishing, polishing, and disinfection

Water sandpaper (grit P500, P1000, P1200) (P.S. Trading, Oltarzew, Poland) and 0.6 mm pumice stone powder (Everall7) were used for finishing the specimens. The upper side of each specimen (or the side made of Durasplint LC for thermoformed specimens) was polished for 1 min with polishing paste for resin and metals (Everall7) using POLIRET Mini (REITEL Feinwerktechnik GmbH, Bad Essen, Germany). Finally, each specimen was rinsed under water and disinfected either by spraying with Incidin Liquid Spray (Ecolab, Krakow, Poland) or by UV irradiation for 30 min on each side using UV-C Blue (Activeshop, Wroclaw, Poland).

### 2.2 Biological evaluation

Both direct and indirect methods were used to assess the biocompatibility of the studied materials. The MTT assay, LDH assay, and wound healing assay were conducted after direct contact with cultured cells. For the PrestoBlue assay, DCF assay, and type of cell death determination, extracts of the materials were added to the cells.

#### 2.2.1 Extract preparation

The extraction procedure followed the ISO 10993-12:2021(E) norm for cytotoxicity testing. Samples were immersed in a cell culture medium (1.57 mL of DMEM (Sigma-Aldrich, St. Louis, MO, United States) in an 8 mL glass vial (Labo24, Gliwice, Poland) to achieve an extraction ratio of 3 cm^2^/mL (surface area/volume). Specimens immersed in DMEM were then extracted for 24 h or 72 h at 37°C with continuous mechanical agitation (100 rpm) in an orbital shaker-incubator (Grant-bio ES-20, Grant Instruments (Cambridge) Ltd., Royston, United Kingdom), following recommendations included in the ISO 10993-12:2021(E) norm.

#### 2.2.2 Cell culture

Human gingival fibroblasts (HGFs) were used for the cytotoxicity testing, as they are predominant cells of the gingival connective tissue, which maintains periodontal tissue homeostasis. Due to their presence in the oral mucosa lamina propria, as well as their multiple biological functions, they serve as a common model for dental materials testing.

The primary cell culture was established following a procedure patented by Dominiak and Saczko (Dominiak and Saczko, 2011), after isolation from 1 to 2 mm fragments of healthy gingival tissue. The study protocol and materials were approved by the Bioethical Committee at Wroclaw Medical University (approval No. 1017/2022).

The HGFs were grown in 75 cm^2^ flasks (Nunc, Roskilde, Denmark) in a humidified atmosphere at 37°C and 5% CO2 in a cell culture medium (DMEM) supplemented with 10% fetal bovine serum (FBS, Sigma-Aldrich) and 1% penicillin/streptomycin (Sigma-Aldrich). After detachment by trypsinization (0.25% Trypsin-EDTA, Sigma-Aldrich), the cells were seeded onto either 24-well cell culture plates (SPL Life Sciences, Gyeonggi-do, Korea) at a concentration of 5 × 10^4^ cells in 500 µL of DMEM/well (for MTT and LDH assays), 96-well black plates with transparent bottoms (SPL Life Sciences) at a concentration of 1 × 10^4^ cells in 100 µL of DMEM/well (for DCF and PrestoBlue assays), or 6-well cell culture plate (SPL Life Sciences) at a concentration of 1.5 × 10^5^ cells in 1,000 µL of DMEM/well (for cell death type assay). The cells were then allowed to attach for 24 hs before the addition of the studied materials or extracts for 24 or 72 hs.

#### 2.2.3 MTT assay

The alterations in mitochondrial function of the cells were evaluated using a 3-(4,5-dimethyl-2-thiazolyl)-2,5-diphenyl-2H-tetrazolium bromide (MTT) assay after 24 or 72 h of direct contact with the materials. The MTT method was used as a basic test recommended for the evaluation of dental materials toxicity according to ISO 10993-5 (Gruber and Nickel, 2023). For this assay, 400 µL of MTT reagent (Sigma-Aldrich) was added to the cell monolayer grown on the 24-well cell culture plate. After 90 min of incubation at 37°C, the formazan crystals were dissolved by mixing with 100 μL of acidic isopropanol. The absorbance was measured at 560 nm using a multiwell plate reader (GloMax Discover Microplate Reader, Promega, Madison, WI, United States). The results were expressed relative to untreated control cells, with normal mitochondrial activity set at 100%.

#### 2.2.4 LDH assay

The level of lactate dehydrogenase (LDH) was measured as an indicator of cytotoxicity, reflecting the release of the cytosolic enzyme into the surrounding cell culture medium due to a damaged plasma membrane. The CyQUANT LDH Cytotoxicity Assay Kit (Thermo Fisher Scientific, Waltham, United States) was used according to the manufacturer’s instructions. Absorbance was measured at 490 and 680 nm using a Multiskan GO microplate reader (Thermo Fisher Scientific). The results were compared to values measured for the positive control (100% of LDH release).

#### 2.2.5 PrestoBlue assay

Cell viability was assessed by monitoring changes in the cellular reducing environment or metabolic activity using a resazurin-based reagent. The PrestoBlue HS cell viability reagent (Thermo Fisher Scientific) was used according to the manufacturer’s protocol. After 30-min incubation of the cells with the reagent, the fluorescence intensity was measured (excitation at 520 nm, emission at 580–640 nm) every 15 min for 90 min using a multiwell plate reader (GloMax Discover Microplate Reader, Promega). The results were expressed relative to untreated control cells, with normal metabolic activity set at 100%.

#### 2.2.6 DCF assay

The carboxy derivative of fluorescein, carboxy-H2DCFDA (Thermo Fisher Scientific) was used as an indicator of reactive oxygen species (ROS) with improved retention within the cell. The assay was conducted following the manufacturer’s protocol. A 10 µM solution of DCF was added to the cells for 30 min. A positive control was prepared using 100 µM H_2_O_2_ (Chempur, Piekary Slaskie, Poland), to induce oxidative activity. After incubation, the fluorescence intensity was measured (excitation at 475 nm, emission at 500–550 nm) every 15 min for 120 min using a multiwell plate reader (GloMax Discover Microplate Reader, Promega).

#### 2.2.7 Type of cell death assay

Dead Cell Apoptosis Kit with Annexin V APC and Sytox Green (Thermo Fisher Scientific) was used to determine the types of cell death induced by incubation of the HGFs with the extracts of the studied materials (obtained after 24 h and 72 h of extraction). Cells incubated with 20 µM staurosporine (Sigma-Aldrich) for 24 h were used as the positive control for apoptosis. After incubation, cells were harvested by trypsinization and centrifuged. The cell pellet was resuspended in PBS(MP Biomedicals, Santa Ana, CA, United States) and stained with allophycocyanin (APC)-conjugated Annexin V (for apoptosis detection) and Sytox Green (for necrosis detection), following the manufacturer’s protocol. After gentle vortexing and incubation for 15 min at 37°C in the dark, the cells were analyzed using a CyFlow CUBE-6 flow cytometer (Sysmex, Poland) equipped with a 633 nm red laser for APC detection and a 488 nm blue laser for Sytox Green detection. For each sample, at least 10,000 events were recorded. The data (fluorescence intensity and scatter properties) were analyzed by flow cytometry software to distinguish live, early apoptotic, apoptotic, and necrotic cells.

#### 2.2.8 Microscopic observations

The cellular morphology of HGFs upon 24 or 72 h of direct contact with the materials was observed under a Leica inverted light microscope (DMi1, KAWA.SKA, Poland) with a ×10 objective.

#### 2.2.9 Cell migration - wound healing assay

Wound healing assay was performed to assess the influence of the studied materials on the cell migration. For this purpose, HGFs (at a concentration of 5 × 10^4^ cells in 70 µL of DMEM) were seeded into a 6-well plate with Ibidi Culture-Inserts 3 Well (Ibidi, Grafelfing, Germany). After 24 h the inserts were removed and two defined cell-free gaps (each of 500 µm) were obtained between three cell monolayers created. Afterward, the studied materials were placed near the cells, and the wells were filled with DMEM (1 mL). Cell culture was observed under a Leica inverted light microscope (DMi1, KAWA.SKA, Poland) at each 12 h between the 0 h and 72 h time points. Software ImageJ (LOCI, University of Wisconsin) with a *Wound Healing Size Tool* plugin was used to quantify the wound closure area (Suarez-Arnedo et al., 2020).

### 2.3 Surface roughness evaluation

The surface profiles of the studied materials were measured using MarSurf PS10 Surface Roughness Measuring Instrument (Mahr, Germany). Mean R_a_ and R_z_ were calculated based on the measurement of 10 specimens for each material. Both unpolished and polished sides were measured in five sites in different directions for each specimen.

### 2.4 Statistical analysis

All measurements were carried out for n ≥ 15 for each group. The results were analyzed using GraphPad Prism 9.1.2 software (GraphPad Software, La Jolla, CA, United States) and were expressed as mean ± SD. Data normality was assessed using the Shapiro-Wilk test. Differences between the cytotoxicity of the analyzed materials and the untreated controls were evaluated using parametric two-way analysis of variance (ANOVA) for multiple comparisons, with posthoc Šídák’s test for LDH and Dunnett’s (for MTT, DCF, and PrestoBlue) multiple comparisons test. Differences in the surface roughness parameters between the analyzed materials were evaluated using parametric ANOVA with posthoc Tukey’s test (for R_a_) and Šídák’s test (for R_z_) for multiple comparisons. Differences were considered statistically significant at p < 0.05.

## 3 Results

### 3.1 Direct contact tests

#### 3.1.1 MTT assay

**FIGURE 3:**
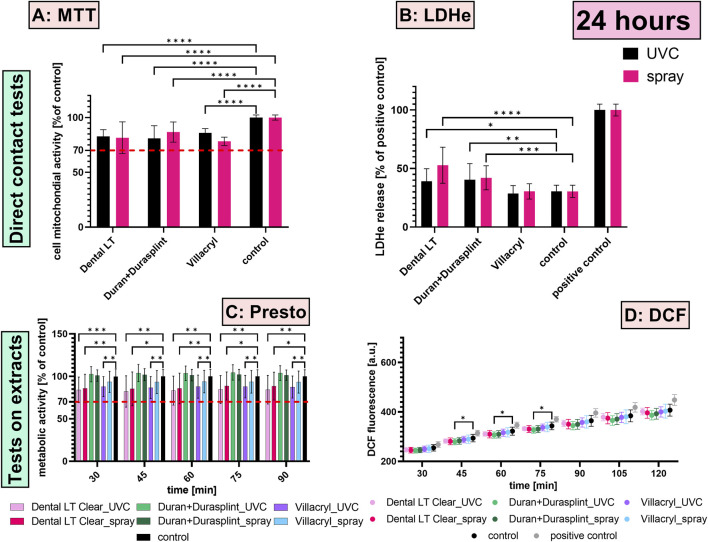
Biological effects measured after 24 h exposition of HGFs to the studied materials via the direct contact (**(A)** MTT assay, **(B)** LDH assay) and the extract tests (**(C)** PrestoBlue assay, **(D)** DCF assay); *p ≤ 0.05, **p ≤ 0.01, ***p ≤ 0.001, ****p ≤ 0.0001. The red dashed line represents 70% of untreated control, being a cut-off level between cytotoxic and non-cytotoxic effects by ISO 10993-5:2009.

**FIGURE 4:**
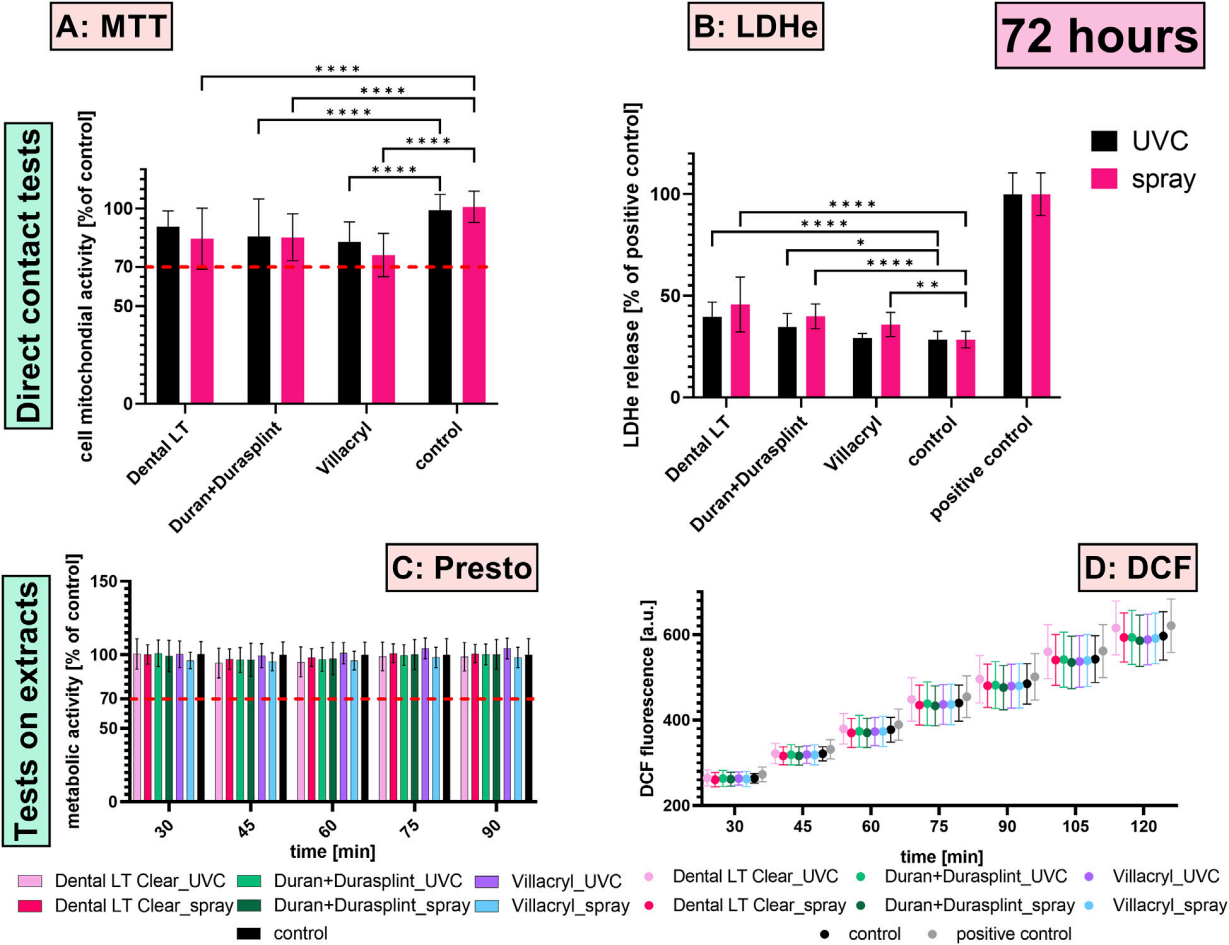
Biological effects measured after 72 h exposition of HGFs to the studied materials via the direct contact (**(A)** MTT assay, **(B)** LDH assay) and the extract tests (**(C)** PrestoBlue assay, **(D)** DCF assay); *p ≤ 0.05, **p ≤ 0.01, ***p ≤ 0.001, ****p ≤ 0.0001. The red dashed line represents 70% of untreated control, being a cut-off level between cytotoxic and non-cytotoxic effects by ISO 10993-5:2009.

The MTT assay revealed a significant decrease in the mitochondrial activity of HGFs after 24 and 72 h of contact with most of the studied materials (Figures 3A, 4A) (p < 0.0001). However, the reduction in metabolic function after 72 h of contact with UV-disinfected Dental LT was not significant (mean = 91%; p = 0.0506). The most substantial decrease was observed for specimens made of heat-cured Villacryl, where mitochondrial activity decreased to 76% of the untreated control after 72 h of contact with chemically-disinfected Villacryl. Despite the statistically significant decrease, all studied materials could be considered safe, as per ISO 10993-5:2009(E), which considers a reduction of cell viability by more than 30% to be a cytotoxic effect.

#### 3.1.2 LDH assay

The highest LDH release was measured for HGFs exposed to Dental LT, with approximately 40%–50% of the positive control for 24 h of contact and 40%–45% for 72 h of contact (Figures 3B, 4B). A slighter effect was detected for Duran+Durasplint, with approximately 40%–42% for 24 h of contact and 35%–40% for 72 h of contact. Comparison between the studied materials and the untreated control revealed that both Dental LT and Duran+Durasplint negatively influenced cell membrane integrity, while Villacryl did not result in a significant increase in LDH release (only the level measured for chemically-disinfected Villacryl after 72 h of contact was significantly higher than for the untreated control; p = 0.0016).

#### 3.1.3 Microscopic observations

**FIGURE 5:**
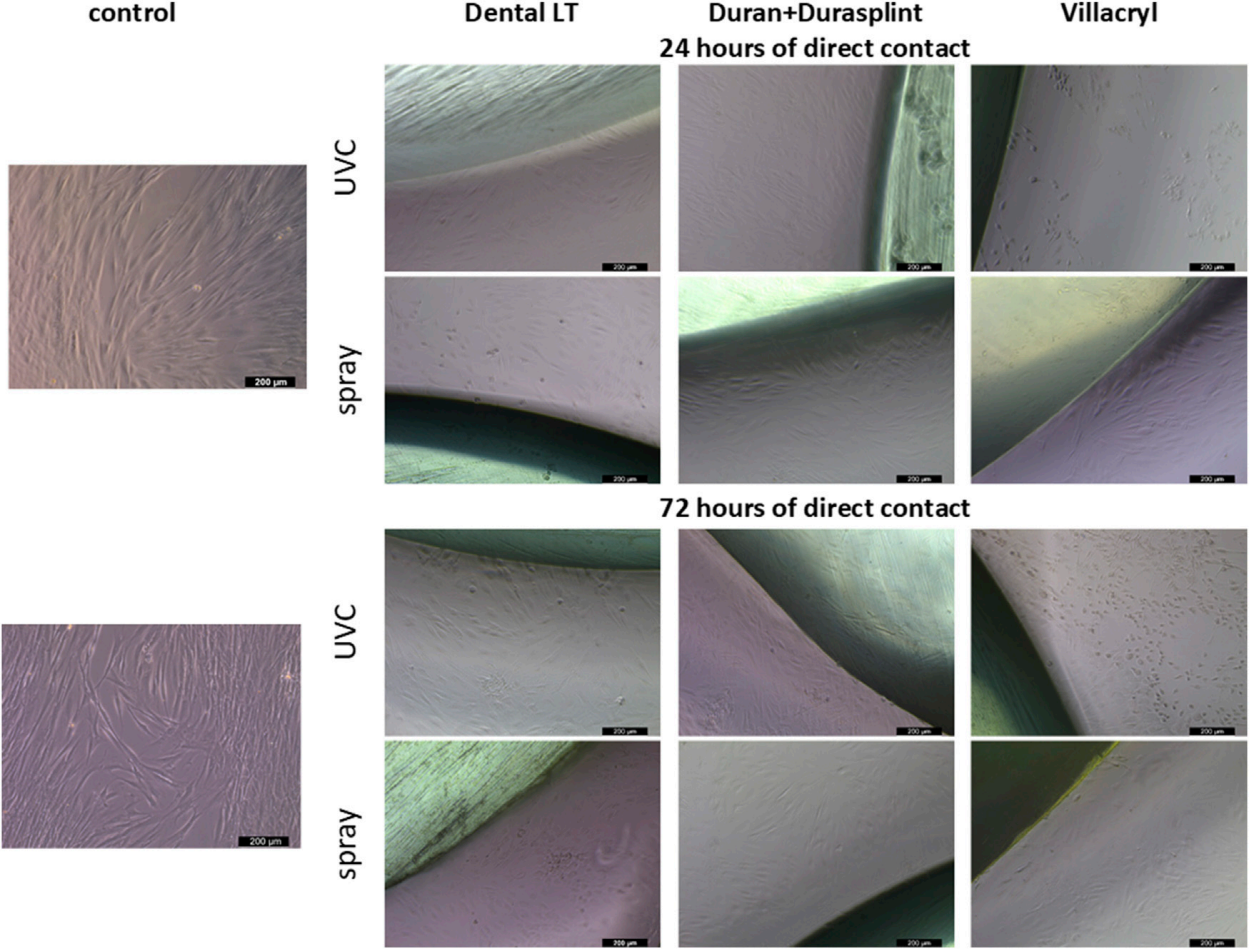
Microscopic images of HGFs exposed to the evaluated materials (observations after 24 h or 72 h of direct contact, magnification: ×100).

Microscopic observations revealed no severe morphological changes in cells after 24 or 72 h of contact with the studied materials (Figure 5). Cells exposed to specimens made of Duran+Durasplint exhibited morphology similar to untreated control cells, with most fibroblasts being tightly attached and elongated. However, cells near the specimens showed changes in morphology, becoming more round and loosely attached. On the other hand, more distant populations of cells had their structure and shape unchanged. Moreover, only slight growth inhibition was observed even after 72 h of contact with the materials.

#### 3.1.4 Cell migration - wound healing assay

**FIGURE 6:**
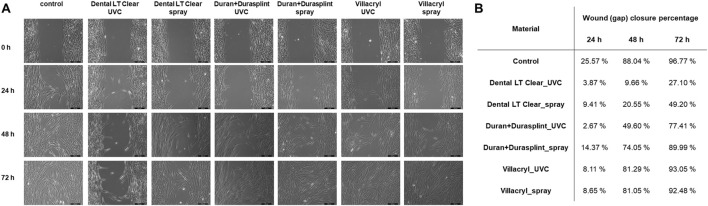
Cell migration evaluated by wound healing assay after exposition of HGFs to the studied materials (**(A)** Microscopic images after 0, 24, 48, and 72 h, **(B)** Wound (gap) closure percentage after 24, 48, and 72 h).

Wound healing assay results revealed that culturing of human gingival fibroblasts in close proximity to the specimens made of Villacryl or Duran+Durasplint did not significantly impair the ability of cells to migrate through the area of gap created in a cell monolayer (Figure 6). For these materials, the wound (gap) closure percentages after 72 h of observation were ca. 80%–90%, which shows that the migration rate was similar to the untreated control cells. However, results obtained for the cells incubated with the specimens made of Dental LT Clear indicate a stronger inhibition of the cell migration, particularly for the material sterilized using UV irradiation, as only ca. 27% of the initial wound (gap) area was closed after 72 h.

### 3.2 Extract tests

#### 3.2.1 PrestoBlue assay

The metabolic activity of HGFs was impaired after 24 h of exposure to the extracts of specimens made of Dental LT and Villacryl (Figures 3C, 4C). On the other hand, extracts of specimens made of Duran+Durasplint did not disturb the mitochondrial function (all p > 0.05). However, none of the reductions measured were considered significant cytotoxic effects, as all values were above 80% of the untreated control. Longer incubation (72 h) resulted in milder effects, with no statistically significant decreases, suggesting cells were able to resume proper functioning and proliferation.

#### 3.2.2 DCF assay

The evaluation of ROS generated upon 24 h exposure of HGFs to the extracts of the materials revealed that they did not induce oxidative stress, as for all the studied groups, the fluorescence of DCF was measured to be lower than that of the untreated control (Figure 3D). Fluorescence in samples incubated with Duran+Durasplint extracts was even significantly lower. After 72 h, the extract of UV-disinfected Dental LT resin had the strongest influence on ROS generation among all materials (Figure 4D). However, the differences from the untreated control were not statistically significant (all p > 0.05).

#### 3.2.3 Type of cell death assay

**FIGURE 7:**
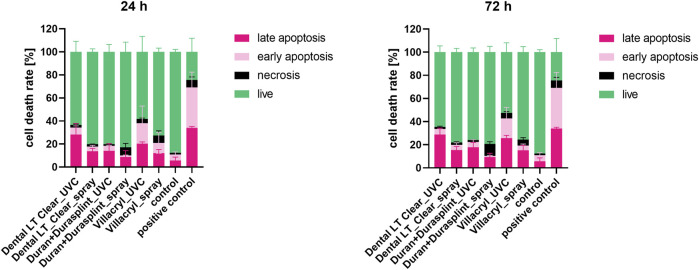
Cell death type measured after 24 h exposition of HGFs to the extracts of the studied materials (obtained after 24 h and 72 h of extraction). Percentages of apoptotic/early apoptotic/necrotic or live cells were detected by flow cytometry.

Flow cytometry analysis shows that the lowest percentage of live cells (52%) was detected after incubation with an extract of Villacryl sterilized by UV (Figure 7). The second most cytotoxic material was Dental LT Clear sterilized by UV (64% of live cells), while for the rest of the samples, more than 70% of cells were viable. For all the materials, apoptosis was predominant over necrosis. The results obtained for the 24 h and 72 h extracts did not differ significantly.

### 3.3 Surface roughness evaluation

The surface roughness parameters of the unpolished materials differ significantly (Table 1). The highest values of both R_a_ and R_z_ were measured for Dental LT Clear, while Duran was characterized by the lowest surface roughness among the unpolished materials. Comparison of the polished specimens revealed a lack of significant differences of both R_a_ and R_z_ between the studied materials.

**TABLE 1:** Surface roughness parameters (Ra and Rz) of the studied materials.

Material	Polishing	R_a_ [µm]		R_z_ [µm]	
		Mean	SD	Mean	SD
Dental LT Clear	no	2.604^A^	0.481	14.923^A^	2.531
Duran	no	0.064^C^	0.038	0.933^C^	0.488
Villacryl	no	1.402^B^	0.602	9.120^B^	3.632
Dental LT Clear	yes	0.176^a^	0.034	1.341^a^	0.293
Durasplint	yes	0.217^a^	0.050	1.797^a^	0.723
Villacryl	yes	0.198^a^	0.076	1.307^a^	0.305

Different capital letters (A, B, C) indicate significant differences between unpolished materials at p < 0.0001, while a small “a” letter indicates lack of significant differences between polished materials at p < 0.05.

## 4 Discussion

The constantly growing demand for various types of intraoral devices creates the necessity of searching for new, cost-saving and material-saving, convenient, and rapid techniques for their fabrication. However, these novel approaches must meet the highest criteria of minimal risk of any adverse effects on the human body. Therefore, further exploration of the biological effects of 3D-printable materials is still necessary (Woźniak-Budych et al., 2023).

In our study, a comparison of the materials revealed that all of them induced a cytotoxic effect in HGFs, both in direct contact tests and when the cells were exposed to the materials’ eluates obtained through extraction in a cell culture medium. However, the decrease in metabolic activity observed was always less than 30% when compared to the untreated control, which is regarded as a lack of cytotoxicity according to ISO 10993-5:2009. Therefore, the null hypothesis of our study cannot be rejected.

The lack of cytotoxicity of additively manufactured, as well as milled and thermoformed materials, was also confirmed by Bürgers et al. (2022). However, some studies reported higher levels of residual monomer in 3D-printed resins than in conventional or milled materials (Wedekind et al., 2021). Another interesting study revealed that Dental LT Clear and Dental SG resins released ovo-toxic leachates, inducing rapid mammalian oocyte degeneration (Rogers et al., 2021). It was also demonstrated that unpolished printed specimens made of Dental LT Clear and Free-print Splint caused cytotoxic effects in HGFs. However, polishing of the specimens enabled the removal of the top layer and significantly improved biocompatibility (Bieger et al., 2023). Due to these inconclusive results, further studies focused on the biocompatibility of dental materials used for 3D printing are still necessary.

One of the strengths of our study is the use of both extract tests and direct-contact tests, as both leachable molecules and the ultrastructure of the resin surface are considered factors mediating the cytotoxic effects of dental materials. Focusing on the role of leachates, oxidative stress and inflammation could be induced by the monomer or unreacted photoinitiator released from the polymeric resin, leading to cell-cycle disorders, a decrease in proliferation, and induction of cell death (Wei et al., 2022; Folwaczny et al., 2023).

Our research provided detailed insight into the cellular effects of the studied materials since two types of metabolic assays were applied: MTT, which is a “gold standard” for testing cytotoxicity, and PrestoBlue, a highly sensitive method measuring the ability of metabolically active cells to reduce resazurin to resorufin enzymatically. Moreover, the evaluation of changes in cell membrane integrity and oxidative stress induced by the exposure of cells to the materials or extracts was also performed. The wound healing assay confirmed the lack of the negative impact of Villacryl and Duran+Durasplint on the cell migration rate. However, reduced cell mobility upon contact of HGFs with Dental LT Clear requires further investigation aimed at the elucidation of the molecular mechanisms affected. Various biological tests were also carried out by Guerrero-Giron´es et al., evaluating differences between conventional resin (Orthocryl) and three various 3D-printed materials. The results of MTT assay, cell migration assay, cell cytoskeleton staining, cell apoptosis, generation of ROS, and scanning electron microscopy confirmed that Orthocryl, Keysplint Soft, and NextDent Ortho Rigid were biocompatible, but Free-print Splint induced cytotoxicity in HGFs (Guerrero-Gironés et al., 2022).

In our study, no significant increase in the accumulation of ROS, which are highly reactive agents able to cause damage to proteins, lipids, and DNA, was detected. This finding might be surprising, as it has been proposed that methacrylate-based monomers induce cytotoxic effects, for example, through increased levels of oxidative stress and concomitant glutathione (GSH) depletion (Juráňová and Jur, 2020). However, one possible explanation could be that proper postprocessing reduces the number of uncured monomers. Several studies have reported that washing and curing strongly influence the final biocompatibility of 3D-printed materials by efficiently removing unreacted monomers (Li et al., 2021; Xu et al., 2021; Lambart et al., 2022; Wulff et al., 2022; Dantagnan et al., 2023; Oh et al., 2023). Interestingly, some alternative methods of post-polymerization treatment, such as autoclaving at 132°C for 4 min, have also been proposed to reduce the elution of the monomer from dental appliances (Tangpothitham et al., 2022; Wuersching et al., 2022).

In order to provide additional material-related insight into the biological effects observed, we decided to compare the surface roughness of the studied materials. This feature has essential clinical implications, as it determines both the comfort of use of the intraoral appliances as well as it strongly influences their microbiological safety. Comparison of the materials selected for our current study revealed that initially Dental LT Clear has the highest R_a_ and R_z_, but, after polishing, all the materials became smoother and no significant differences of the surface roughness parameters were reported. As a mean R_a_ of 0.2 μm is treated as the critical threshold for bacterial plaque retention, in our study the values obtained after polishing of the materials may be considered as acceptable for safe clinical applicability (Miyashita-Kobayashi et al., 2024).

### 4.1 Limitations and perspectives

The tests performed in our study focused on evaluating the effects of substances released from the specimens by passive hydrolysis, neglecting the influence of intraoral factors on resin degradation. Further studies should consider the effects of the complex oral environment, including temperature and pH changes, salivary enzymes, microbial activity, and functional and parafunctional loads (e.g., masticatory forces, teeth grinding), on the final biocompatibility of intraoral devices. In our previous study, we compared the mechanical properties of Dental LT Clear, Duran+Durasplint and Villacryl, It was demonstrated that the resin for 3D printing had the highest Shore D hardness among all non-aged materials, while the conventional heat-curing material possessed superior flexural properties (Weżgowiec et al., 2024). Moreover, Villacryl had the highest resistance to artificial aging and it was concluded that currently, the conventional material still seems to be the best choice for intraoral appliances intended for long-term use. In light of the results related to the mechanical behavior of the materials, understanding the mechanisms of cytotoxicity due to short-term release should also be accompanied by analyses of long-term effects related to degradation over time, simulated by various modalities of artificial aging.

A second limitation is the number of materials under investigation. Only one material of each type was compared, so the results cannot be generalized to all 3D-printed resins currently available. Moreover, the use of simplified specimens may not fully represent the behavior of intraoral appliances with complex shapes. Finally, in addition to assessing cytotoxicity, a chemical study would also be beneficial in determining the release of substances from the materials.

### 4.2 Conclusions

Based on the findings of this *in vitro* study, the following conclusions can be drawn:

• All evaluated materials demonstrated biocompatibility for short-term intraoral use.
• 3D printing appears to be a safe alternative to currently used methods for intraoral appliances.
• Further tests assessing the long-term effects of these materials are necessary.

## Data Availability

The raw data supporting the conclusions of this article will be made available by the authors, without undue reservation.
